# Incidence and Prevalence of Tuberculosis among Household Contacts of Pulmonary Tuberculosis Patients in a Peri-Urban Population of South Delhi, India

**DOI:** 10.1371/journal.pone.0069730

**Published:** 2013-07-26

**Authors:** Jitendra Singh, Manimuthu Mani Sankar, Sandeep Kumar, Krishnamurthy Gopinath, Niti Singh, Kalaivani Mani, Sarman Singh

**Affiliations:** 1 Division of Clinical Microbiology and Molecular Medicine, All India Institute of Medical Sciences, New Delhi, India; 2 Lala Ram Sarup Institute of Tuberculosis and Respiratory Diseases, New Delhi, India; 3 Department of Biostatics, All India Institute of Medical Sciences, New Delhi, India; Institute of Infectious Diseases and Molecular Medicine, South Africa

## Abstract

**Background:**

Tuberculosis (TB), caused by *Mycobacterium tuberculosis*, is one of the leading causes of mortality and morbidity across all age groups throughout the world, especially in developing countries.

**Methodology/Principal Findings:**

In this study, we have included 432 open index cases with their 1608 household contacts in a prospective cohort study conducted from May 2007 to March 2009. The follow-up period was 2 years. All Index cases were diagnosed on the basis of suggestive signs and symptoms and sputum being AFB positive. Among the 432 index patients, 250 (57.9%) were males and 182 (42.1%) females; with mean age of 34±14.4 yr and 26±11.1 yr, respectively. Out of 1608 household contacts, 866 (53.9%) were males and 742 (46.1%) females; with mean age of 26.5±15.8 and 26.5±16.0 yr, respectively. Of the total 432 households, 304 (70.4%) had ≤4 members and 128 (29.6%) had ≥5 members. The median size of the family was four. Of the 1608 contacts, 1206 were able to provide sputum samples, of whom 83 (6.9%) were found MTB culture positive. Household contacts belonging to adult age group were predominantly (74, 89.2%) infected as compared to the children (9, 10.8%). On screening the contact relationship status with index patients, 52 (62.7%) were first-degree relatives, 18 (34.6%) second-degree relatives and 12 (14.5%) spouses who got infected from their respective index patients. Co-prevalent and incident tuberculosis was found in 52 (4.3%) and 31 (2.6%) contacts, respectively. In incident cases, the diagnosis could be made between 4 to 24 months of follow-up, after their baseline evaluation.

**Conclusion:**

Active household contact investigation is a powerful tool to detect and treat tuberculosis at early stages and the only method to control TB in high-TB-burden countries.

## Introduction

Tuberculosis (TB), caused by *Mycobacterium tuberculosis*, is one of the leading causes of mortality and morbidity across all age groups throughout the world, especially in developing countries. In 2011, there were an estimated 8.7 million incident cases of TB (range, 8.3 million–9.0 million) globally and equivalent to 125 cases per 100 000 population [1]. Most of the estimated number of cases in 2011 occurred in Asia (59%) and Africa (26%); India and China alone accounted for 26% and 12% of global cases, respectively [1].

The infection is almost exclusively transmitted through air from patients with pulmonary disease. The risk of transmission to household contacts is greatest when index case is sputum smear positive, closeness of the index case with contacts, overcrowded living conditions, bacillary density in respiratory secretions, and degree of lung fields involved [2]–[5]. Therefore, those living within the same household are at higher risk than casual contacts [6]–[7]. Further, among the household contacts, younger age and absolute or relative immunodeficiency states are at higher risk of acquiring infection from their index case [8]–[9]. Contact investigation for cases of active pulmonary TB is standard practice in developed countries [10]. Several other studies from high burden countries have shown that active case finding among household contact yields significantly more TB cases than passive case detection [11]–[17].

Government of India envisions for a “TB-free India”, by 2017, which aims to reduce the burden of TB until it is no longer a major public health problem [18]. To achieve this vision, national tuberculosis control program must emphasize on early diagnosis and treatment of patients to minimize the risk of disease transmission to their contacts and vulnerable population. As of now only passive case detection is in place, in which the symptomatic patient comes to the microscopy center and if found AFB positive, he/she is administered with anti-tubercular treatment. However, to make TB-free India, active case finding is extremely crucial. For this, not only symptomatic cases (index cases) but co-prevalent and incident cases of TB should also be tracked, diagnosed and treated at the earliest. Several studies have found contact investigation to be a very efficient method of identifying asymptomatic TB cases [19]–[22], but unfortunately not many systematic studies are conducted in India, on this important aspect. Although exposed contacts are usually sputum smear negative and do not contribute to the immediate spread of the disease, they form a pool of infection from which a significant number of future index cases will arise. Therefore, in recent years, contact tracing has started gaining significance and it is incorporated into the Revised National Tuberculosis Control Programme (RNTCP) of the Government of India [23]–[24].

This study was undertaken in a cohort from a peri-urban area of Delhi, India, aiming to find the prevalence of open pulmonary tuberculosis and detect the disease in their household contacts before they become symptomatic. We also tried to identify possible risk factors for transmission and the timing of development of symptomatic TB in household contacts. Further, we also analyzed the bacteriological methods of early detection of index and household contacts and factors associated with co-prevalent and incident tuberculosis in the cohort.

## Results

**Figure 1:**
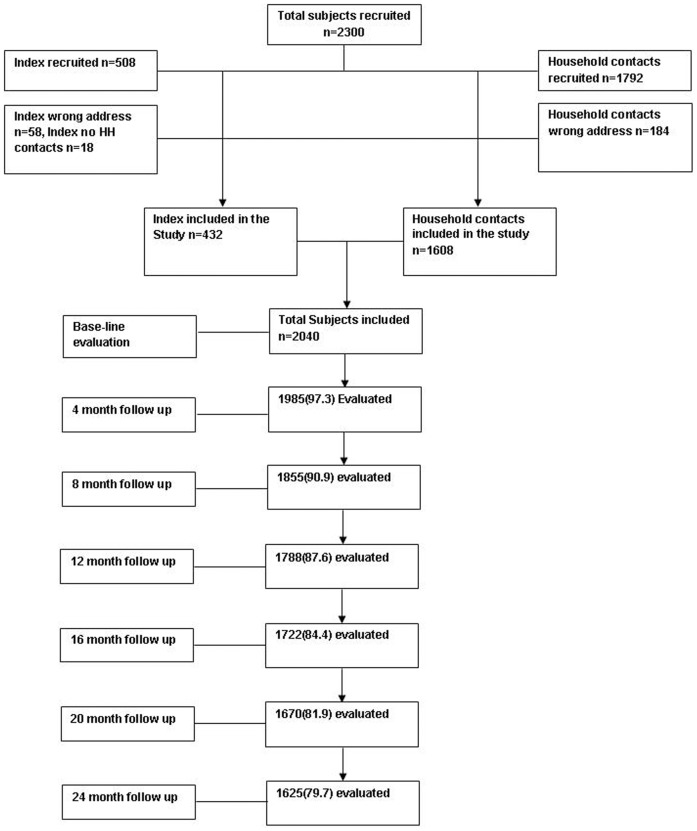
Flow chart showing details of recruitment of subjects and their follow up at every four months.

**Figure 2:**
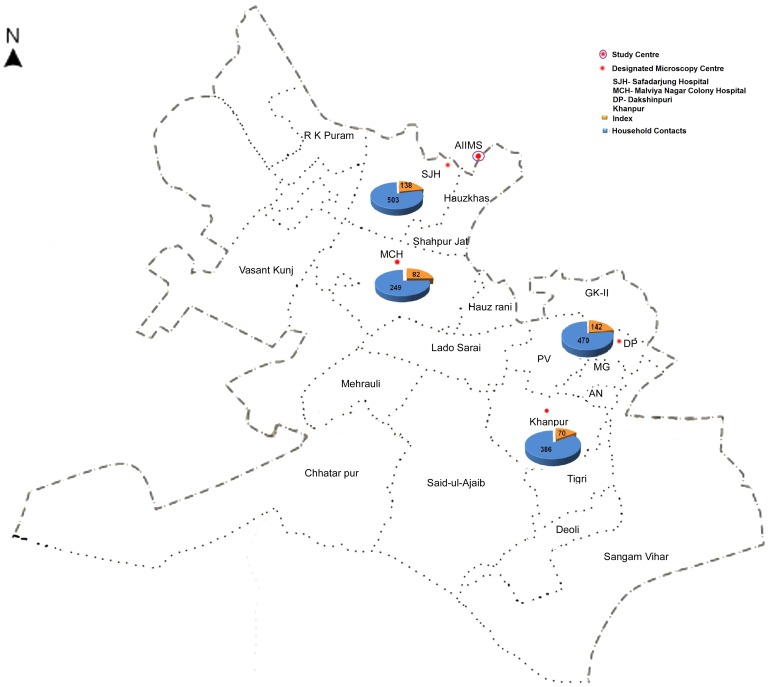
Map of South Delhi DOTS Centers and their surrounding areas from where all the index cases and their household contacts were recruited.

Total duration of the study was 4 years, from May 2007 to May 2011. This included 2 year enrolment period (May 2007–May 2009) and 2 years follow-up period (upto May 2011). Between May 2007 to May 2009, a total of 508 open index cases and their 1792 household contacts were enrolled from various Directly Observed Treatment Short- course (DOTS) centers of South Delhi region. Of these, only 432 index cases and 1608 contacts were included in the study, while the remaining were excluded for various reasons (**Figure 1**). Majority of index cases were from Dakshinpuri, while most contacts were from Safardarjung area (**Figure 2**).

Among the 432 index patients, 250 (57.9%) were males and 182 (42.1%) females, with mean age of 34±14.4 and 26±11.1 years, respectively. Majority of the index patients were adults (426, 98.6%) and only 6 (1.4%) aged <12 years. The median size of family was four members; with 304 (70.4%) households having ≤4 members and remaining 128 (29.6%) had ≥5 members. A total of 372 (86.1%) index cases were residing in highly crowded or congested surrounding with poor sanitation in their locality and remaining 60 (13.9%) resided in comparatively clean surroundings. It was observed that 395 (91.4%) households were living in houses with ≤2 rooms and 37 (8.6%) in houses of 3–4 rooms (**Table 1**). On clinical examination, cough was predominant manifestation (94.7%), followed by fever (91%), weight loss (63.2%), anorexia (69%) and chest pain (**Table 1**). BCG scar was found in 351 (81.3%) patients. PPD skin test could be performed only in 418 patients; of whom 409 (97.9%) patients showed positive reaction (10×10 mm).

**Table 1:** Demographical and Clinical details of Index and Household Contacts.

Characteristics	Index cases, N = 432	Total Household contacts, N = 1608	Household healthy contacts, N = 1525	Household contacts with Co-prevalent TB, N = 52	Household contacts with Incident TB, N = 31
Gender					
Male	250 (57.9)	866 (53.9)	826 (54.2)	24 (46.2)	19 (61.3)
Female	182 (42.1)	742 (46.1)	699 (45.8)	28 (53.8)	12 (38.7)
Mean Age (years ± SD)	31±13.9	26.5±15.9	26.2±15.9	33.8±16.8	26.1±14.1
Age (in years)					
≤12	6 (1.4)	309 (19.2)	300 (19.7)	5 (9.6)	4 (12.9)
13–24	181 (41.9)	552 (34.3)	526 (34.5)	11 (21.2)	15 (48.4)
25–40	44 (3.3)	429 (26.7)	403 (26.4)	19 (36.5)	7 (22.6)
>40	101 (23.4)	318 (19.8)	296 (19.4)	17 (32.7)	5 (16.3)
Relationship					
Spouse	-	200 (12.4)	188 (12.3)	10 (19.2)	2 (6.5)
I^st^ degree relative	-	1154 (71.8)	1101 (72.2)	33 (63.5)	20 (64.5)
II^nd^ degree relative	-	254 (15.8)	236 (15.5)	9 (17.3)	9 (29.0)
Previous self history of TB					
Yes	127 (29.4)	12 (0.8)	10 (0.7)	1 (1.9)	1 (3.2)
No	305(70.6)	1596 (99.2)	1515 (99.3)	51 (98.1)	30 (96.7)
BCG vaccination (n = 1329)					
Yes	351 (81.3)	1160 (87.3)	1100 (87.8)	38 (84.4)	22 (71.0)
No	81 (18.7)	169 (12.7)	153 (12.2)	7 (15.6)	9 (29.0)
PPD skin test					
Positive (≥10 mm)	409 (97.9)	1172 (84.4)	1094 (83.7)	37 (78.7)	27 (87.1)
Negative (<10 mm)	9 (2.1)	217 (15.6)	213 (16.3)	10 (21.3)	11 (12.9)
Household Size					
Household with 2–4 members	304 (70.4)	845 (52.6)	806 (52.9)	30 (57.7)	9 (29.0)
Household with 5–7 members	114 (26.4)	630 (39.2)	600 (39.3)	18 (34.6)	12 (38.7)
Household with ≥8 members	14 (3.2)	133 (8.2)	119 (7.8)	4 (7.7)	10 (32.3)
No. of room/Household					
Household with 1–2 room	395 (91.4)	1431 (89.0)	1355 (88.9)	48 (92.3)	28 (90.3)
Household with 3–4 room	37 (8.6)	177 (11.0)	170 (11.1)	4 (7.7)	3 (9.7)
Household Environment					
Congested/Semi-urban	372 (86.1)	1375 (85.5)	1298 (85.1)	50 (96.1)	27 (87.1)
Clean	60 (13.9)	233 (14.5)	227 (14.9)	2 (3.9)	4 (12.9)
Fever					
Yes	393 (91.0)	40 (2.5)	0	20 (38.5)	20 (64.52)
No	39 (9.0)	1568 (97.5)	1525 (100.0)	32 (61.5)	11 (35.48)
Cough					
Yes	409 (94.7)	59 (3.7)	0	30 (57.7)	29 (93.6)
No	25 (5.3)	1549 (96.3)	1525 (100.0)	22 (42.3)	2 (6.6)
Anorexia					
Yes	298 (69.0)	22 (1.4)	0	17 (32.7)	5 (16.1)
No	134 (31.0)	1586 (98.6)	1525 (100.0)	35 (67.3)	26 (83.9)
Breathlessness					
Yes	84 (19.4)	14 (0.9)	0	8 (15.4)	2 (6.5)
No	348 (80.6)	1594 (99.1)	1525 (100.0)	44 (84.6)	29 (93.6)
Weight loss					
Yes	273 (63.2)	28 (2.9)	0	15 (28.9)	13 (41.9)
No	159 (36.8)	1580 (97.1)	1525 (100.0)	37 (71.1)	18 (58.1)
Night Sweats					
Yes	135 (31.2)	14 (0.9)	0	7 (13.5)	7 (22.6)
No	297 (68.8)	1594 (99.1)	1525 (100.0)	45 (86.5)	24 (77.4)
Fatigue					
Yes	357 (82.6)	33 (2.1)	0	18 (34.6)	15 (48.4)
No	75 (17.4)	1575 (97.9)	1525 (100.0)	34 (65.4)	16 (51.6)
Chest pain					
Yes	209 (48.4)	14 (0.9)	0	7 (13.5)	7 (22.6)
No	223 (51.6)	1594 (99.1)	1525 (100.0)	45 (86.5)	24 (77.4)

Values given in parenthesis are percentage.

Of the 1608 contacts, 866 (53.9%) were males and 742 (46.1%) females with mean age of 26.5±15.8 years and 26.5±2 years, respectively. Most of them were adults. On baseline recruitment when we asked the contacts for prevailing symptoms; we found that 40 (2.5%) contacts gave affirmative answer for fever, cough [59 (3.7%)], fatigue [33 (2.1%)] and previous history of TB infection [12(0.8%)]. BCG scar was found in 1160 (87.3%) contacts. PPD testing could be performed only in 1389 contracts, of whom 1172 (84.4%) had positive reaction. The biological relationship of contacts with their index cases showed that 1154 (71.8%) were I^st^ degree relatives, 254 (15.8%) II^nd^ degree relatives and 200 (12.4%) spouses (**Table 1**).

**Figure 3:**
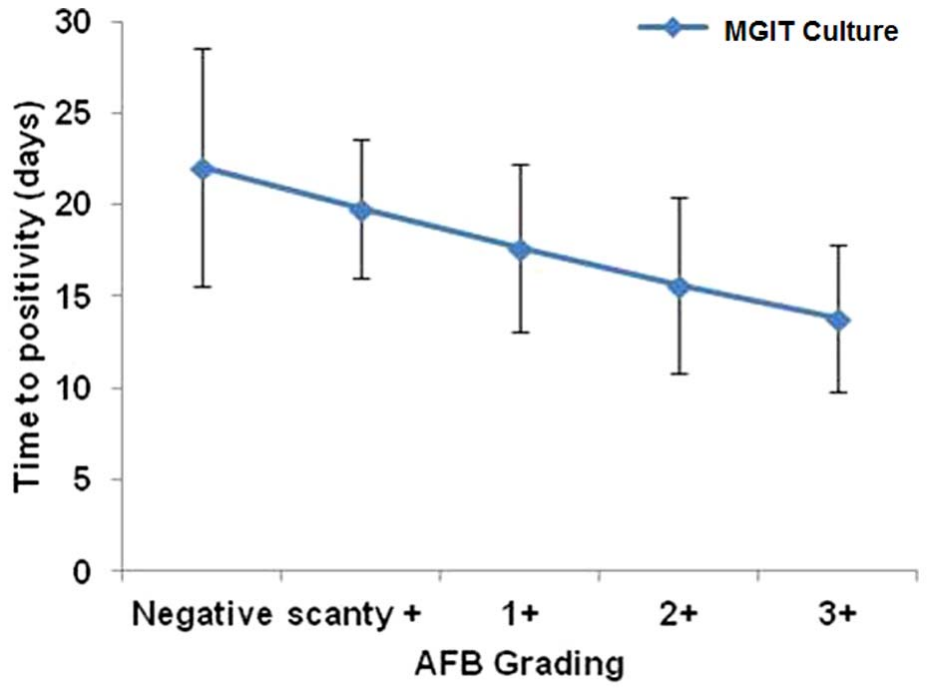
Time to positivity (TTP) in liquid cultures and its correlation with smear positivity.

All the sputum samples from 432 index cases and 1206 contacts, who could produce sputum, were bacteriologically tested, of whom, 408 index cases and 83 contacts (6.8%) were bacteriologically positive while 24 index cases and 1123 contacts were culture negative. Of the 83 secondary tuberculosis cases, 52 (62.7%) had co-prevalent tuberculosis and 31 (37.3%) had incident tuberculosis (**Table 2**). The time to positivity (TTP) for 3+ AFB positive samples was 13.8 days while for 2+, 1+ and scanty positive samples the TTP was 15.6, 17.6 and 19.8 days, respectively. For AFB negative samples, the TTP took 22 days (**Figure 3**). During the periodic follow-up of index cases, mortality was observed in 41 cases of which 31 (6.9%) were due to TB and rest died of other reasons. Of the TB related deaths 19 (61.3%) were males and 12(38.7%) females. Among the deceased cases, three (9.6%) were category I (Cat I) treatment defaulters, nine (29%) non-responders to ATT, 12 (38.7%) relapse cases and seven (22.5%) were failures to category II anti-tubercular treatment.

**Table 2:** Bacteriological details of tuberculosis cases.

Diagnosis	Index cases N = 432	TB in household contacts N = 83		
		Co-prevalent TB N = 52 (62.7)	Incident TB N = 31 (37.3)	*p*-value
ZN staining				
Scanty+	34 (7.9)	1 (1.9)	6 (19.3)	
1+	92 (21.3)	6 (11.5)	3 (9.7)	
2+	71 (16.4)	1 (1.9)	0	0.032
3+	110 (25.5)	0	0	
Negative	125 (28.9)	44 (84.6)	22 (71.0)	
Culture positive*	408 (94.4)	52	31	
Bacteriology Identification				
ZN +, Culture +	307 (71.1)	8 (15.4)	9 (29.0)	0.136
ZN −, Cultural +	101 (23.4)	44 (84.6)	22 (71.0)	
ZN −, Culture −	24 (5.6)	0	0	

* Culture positive by BACTEC™ MGIT 960. Values given in parenthesis are percentage. ZN: Ziehl-Neelsen Staining; +, Positive; −, Negative.

**Figure 4:**
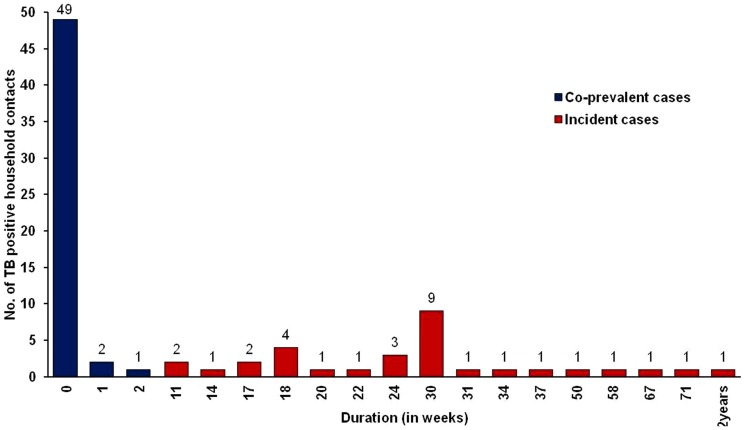
Time of detecting co-prevalent (Cp) and incident (Ip) secondary tuberculosis cases.

All 83 secondary tuberculosis cases could be traced to 62 (14.4%) families with at least 1 primary index case in each family. In one family as many as six contacts fell ill of secondary tuberculosis, of which 1 contact had co-prevalent TB and 5 had incident TB which was detected during the follow-up visits (**Figure 4** **)**. Upon cluster analysis of 62 families, the co-prevalent and incident TB in household contacts were 32.3% (95% CI: 23.1–41.5) and 19.9% (95% CI: 0.76–32.2), respectively. The risk of co-prevalent TB in a household contacts increased with age of the contact when analyzed using univariate or multivariate analyses. The proportion of TB cases was lower in BCG-vaccinated as compared to non-vaccinated contacts but the difference was not statistically significant (p = 0.963). The environment seemingly played major role in transmission of TB from index cases to household contacts. Accordingly, contacts and index cases residing in a congested living area were more likely to acquire TB from their index (p = 0.065) patient compared to the residents of clean environment (**Table 3**).

**Table 3:** Factors associated with co-prevalent tuberculosis cases among household contacts of index cases.

Characteristics	Positive household contacts (%)	Negative household contacts (%)	Crude (95% CI)	*p* value	Adjusted (95% CI)	*p* value
Age						
≤12	5 (9.6)	300 (19.7)	1.0		1.0	
13–24	11 (21.2)	526 (34.5)	1.2 (0.3–4.3)	0.740	0.7 (0.1–4.5)	0.791
25–40	19 (36.5)	403 (26.4)	2.8 (0.8–8.9)	0.078	1.9 (0.3–10.6)	0.458
>40	17 (32.7)	296 (19.4)	3.4 (1.0–11.1)	0.040	3.6 (0.5–23.3)	0.175
Gender						
Male	28 (53.8)	826 (54.2)	1.0		1.0	
Female	24 (46.2)	699 (45.8)	0.9 (0.5–1.6)	0.999	1.9 (0.6–2.1)	0.565
BCG						
Negative	7 (15.6)	153 (12.2)	1.0		1.0	
Positive	38 (84.4)	1100 (87.8)	0.7 (0.3–1.8)	0.570	0.9 (0.3–2.8)	0.963
Mantoux test						
Negative	10 (21.3)	342 (31.1)	1.0		1.0	
Positive	37 (78.7)	758 (68.9)	1.6 (0.7–3.5)	0.185	1.2 (0.5–3.1)	0.611
Environment						
Clean	2 (3.9)	231 (14.9)	1.0		1.0	
Congested	50 (96.1)	1325 (85.1)	4.3 (1.0–17.6)	0.039	6.6 (0.8–49.8)	0.065
Relationship						
Spouse	10 (19.2)	188 (12.3)	1.0		1.0	
I^st^ degree relative	33 (63.5)	1121 (72.0)	0.5 (0.2–1.2)	0.123	0.9 (0.3–2.3)	0.872
II^nd^ degree relative	9 (17.3)	245 (15.7)	0.6 (0.3–1.7)	0.449	1.0 (0.3–3.3)	0.931
Individual person/Room						
≤2	18 (34.6)	482 (31.0)	1.0		1.0	
>2	34 (65.4)	1074 (69.0)	0.8 (0.4–1.5)	0.593	0.9 (0.4–1.9)	0.857

Values given in parenthesis are percentage.

**Figure 5:**
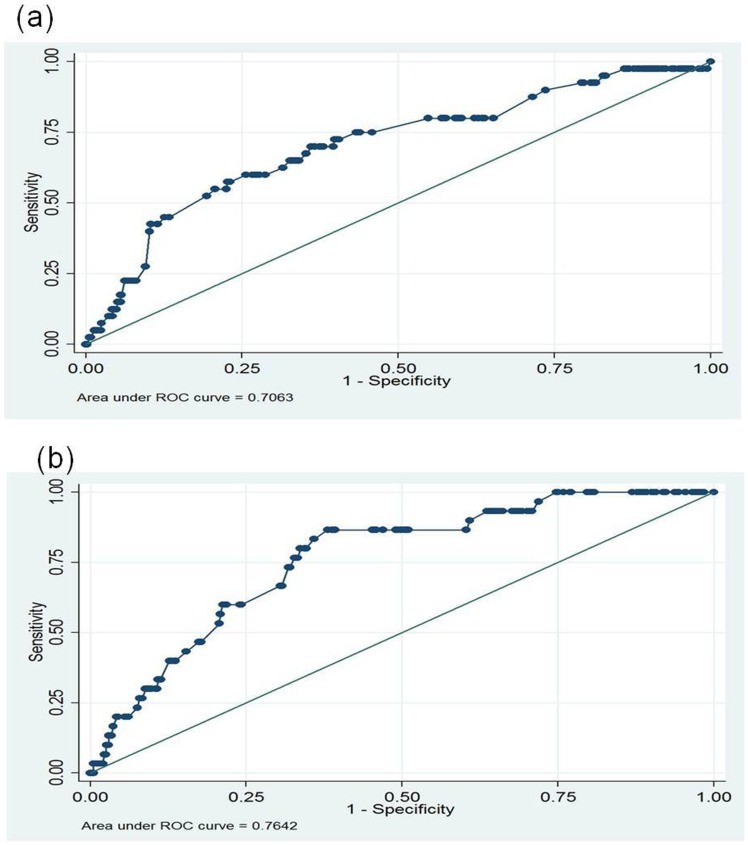
Receiver Operating Characteristic (ROC) curves for Co-prevalent (a) and Incident TB (b) cases.

Of the 31 incident TB cases, diagnosis in them could be established between 4 to 24 months after baseline investigation (**Figure 4**). Univariate logistic regression analysis showed that the risk of incident TB decreased with the advancement of age (OR: 0.3 95%CI 0.7–1.8) (**Table 4**). We also analyzed the transmission rate from index to their household contacts based on ATT category of treatment. It was found that out of 305 index patients prescribed with category I treatment, 51 (16.7%) of their contacts acquired TB. In contacts of index patients on category II treatment (127), a significantly higher percentage [32 (25.2%)] got infected with TB. ROC analysis showed that area under curve (AUC) for co-prevalent cases were 70.6% and for incident tuberculosis cases it was 76.4% [**Figure 5 (a) and 5 (b)**]. This difference between co-prevalent and incident cases was not significant.

**Table 4:** Factors associated with incident tuberculosis cases among household contacts of index cases.

Characteristics	Positive household contacts (%)	Negative household contacts (%)	Crude (95% CI)	*p* value	Adjusted (95% CI)	*p* value
Age						
≤12	4 (12.9)	300 (19.6)	1.0		1.0	
13–24	15 (48.4)	526 (34.5)	2.1 (0.7–5.9)	0.142	0.9 (0.3–2.6)	0.918
25–40	7 (22.6)	403 (26.8)	1.3 (0.5–3.3)	0.578	0.5 (0.1–1.7)	0.287
>40	5 (16.1)	296 (19.4)	1.2 (0.4–3.9)	0.682	0.3 (0.7–1.8)	0.226
Gender						
Female	19 (61.3)	699 (45.8)	1.0		1.0	
Male	12 (38.7)	826 (54.2)	0.5 (0.3–0.9)	0.039	0.5 (0.2–1.1)	0.144
BCG						
Negative	9 (29.0)	153 (12.2)	1.0		1.0	
Positive	22 (71.0)	1100 (87.8)	0.3 (0.1–0.7)	0.004	0.1 (0.03–0.3)	0.001
Mantoux test						
Negative	11 (36.7)	331 (30.9)	1.0		1.0	
Positive	19 (63.3)	739 (69.1)	0.7 (0.3–1.6)	0.495	0.8 (0.3–1.8)	0.683
Environment						
Clean	4 (12.9)	227 (14.9)	1.0		1.0	
Congested	27 (87.1)	1298 (85.1)	1.1 (0.2–6.4)	0.848	1.0 (0.1–6.7)	0.955
Relationship						
Spouse	2 (6.5)	188 (12.3)	1.0		1.0	
I^st^ degree relative	20 (64.5)	1101 (72.2)	1.7 (0.3–7.6)	0.483	1.5 (0.2–8.4)	0.592
II^nd^ degree relative	9 (29.0)	245 (15.5)	3.5 (1.0–11.7)	0.035	3.6 (1.1–12.2)	0.033
Individual person/room						
≤2	7 (22.6)	475 (31.1)	1.0		1.0	
>2	24 (77.4)	1050 (68.8)	1.5 (0.4–4.8)	0.452	1.5 (0.4–5.1)	0.462

Values given in parenthesis are percentage.

BCG vaccinated contacts had lower risk of TB acquisition than the non-vaccinated contacts (**Table 1** **&** **3**). Among various relations and risk of TB infection in household contacts from index cases, 2^nd^ degree relatives had a highest risk (adjusted OR: 3.6, 95% CI: 1.1–12.2) of TB infection, but this risk was closely associated with crowded living conditions.

## Discussion

In recent years, with more funding devoted to TB control and the threat of multi and extensively drug resistant TB (MDR, XDR-TB) [25] contact tracing and active case finding strategies have gained much significance. Guidance for contact investigation is provided in selected policy documents such as British Thoracic society [26] and National Institute of Health and Clinical Excellence [27] but these are generally for use in low TB incidence and high resource settings. The World Health Organization (WHO) has not issued any clear guidance on how to conduct contact investigation or how to prioritize contacts except to say that children <5 years of age and persons with HIV infection are high-priority groups for contact tracing.

India, which is a TB high burden country, does not have detailed guidelines for contact investigation and the procedures for contact investigations have not been standardized at a national level. Consequently the contact investigations are largely dependent on local understanding and practices. From India only 6 studies have been published [11]–[13], [16]–[18]; of these 3 were from south India [11], [12], [18] and 3 from northern India. The authors of all these studies did only microscopic examination of the index cases and their contacts and none used culture methods for the detection of tuberculosis. Hence this was our greatest strength. Our study clearly shows that a highly significant number of co-prevalent (84.6%) and incident tuberculosis (70.9%) were smear negative. This means that only 1 in 5 cases are detected if only smear AFB examination is undertaken. Hence these undiagnosed co-prevalent cases could be responsible for transmitting the infection to other household or other contacts. Moreover, these studies were carried out several decades back e.g. 1961 [11], 1966 [12], 1984 [13], 2004 [16], and 2005 [17]. Only one study was recent [18] and in most of these studies only a small number of index cases were included. While in our study we scrupulously prepared protocol and this instrument was field tested before applying to the study. Moreover, we recorded clinico-demographic details, their vaccination history and other details meticulously. Ours was also the first study to subject all sputum samples for MGIT960 cultures and all subjects (both index cases and household contacts) were offered mantoux test. The size of the cohort was largest in our study. All *M. tuberculosis* isolates were fully characterized and their drug susceptibility pattern determined (details not shown here).

In our study, all index cases were recruited after being reported AFB positive from designated microscopy centers. We observed that 24 (5.6%) index cases were smear negative in our laboratory. This could be due to the fact that after being reported AFB positive by the RNTCP's microscopy centers, these patients were prescribed with anti-tubercular treatment immediately and we could trace these patients and collect samples after few days but within 15 days, as per our study protocol. This finding indicates that upto 5.6% open PTB cases can convert to the smear negativity within 15 days of ATT and do not carry risk of transmitting the disease to contacts. This was further evident from the fact that no contact from the families of these index cases developed secondary tuberculosis. The TTP obtained in this study with regard to AFB load, it was comparable to our previous study [28]. Here, sputum samples from 1206 contacts were cultured for TB and overall we identified 6.9% of household contacts were culture positive for *M. tuberculosis*.

We prospectively evaluated the risk and rate of transmission among household contacts residing with open index patients. While most of the household contacts were asymptomatic at the time of baseline investigation, the clinical signs and symptoms were variable when tuberculosis could be detected during follow-up visits. In bacteriologically (culture positive) confirmed household contacts (n = 83) these manifestations included cough (71.1%), fever (48.2%), fatigue (39.7%), weight loss (33.7%) and night sweats(16.9%) (**Table 1**). These clinical observation of close contacts could be used as high risk index of clinical markers for contact tracing in a given setting. But PPD skin testing of the household contacts was not useful in early detection of tuberculosis as it lacked specificity (p = 0.491). This was probably due to its high sensitivity to *M. tuberculosis* exposure from the contacts, even though the infection could not manifest in disease form during the limited 2 years follow-up.

Various authors have reported varying prevalence rates of pulmonary tuberculosis by age in household contacts of TB index patients [11], [13]–[14], [16]–[24]. We also found this variability in different age groups where that middle aged and elderly persons were more vulnerable for acquiring secondary tuberculosis than children. Further we found that male contacts had considerably higher (51.8%) risk of having co-prevalent TB than female contacts (48.2%). Unsurprisingly, most household contacts (80.8%) were adults. This could be due to urbanization and migration of the working (adult) population to this residential area of Delhi for employment. As the study site is hugely populated and congested, a total of 1431 (89%) of contacts live with open index cases in households with one or two room accommodations. Most of these families have several co-habitants. The maximum number of members in a single family was thirteen. Contacts often shared the same bedroom or the same bed with the index case. This intact and close living with open index patients increases the risk of disease transmission. Though, we shall be able to link these infections in contacts only when our genotypic data is fully analyzed, yet it was very clear that the rate of secondary tuberculosis was 6.8% in the household contacts, studied in present study, which was higher than the Indian average of incident TB cases (0.075%). This finding was in concordance with previous observation, where it is reported that secondary tuberculosis was 10 to 60 times more likely in contacts than the general population [29].

We detected most of the secondary tuberculosis cases at the time of baseline household evaluation but some incident TB cases were also detected during the follow up visits. During the base-line investigation, we faced a great challenge to differentiate between co-prevalent secondary tuberculosis cases in household contacts and primary index cases. In an illiterate population, when two patients have fallen ill, it may be preferential for such families to take the bread earner, usually a male, to the clinic, even if the other family member is more sick for a longer duration [30]. Though this remains a major limitation of any study in such a poor population, our criteria was to consider index case, a patient who first reported to the microscopy center and was smear positive. However, we found that using this recruitment criteria, only a handful of co-prevalent (<15%) cases could be misdiagnosed as index cases. Hence the recruitment criteria we adopted, was most suitable for such a field study. The RNTCP also needs to consider the liquid culture based case detection, after this study.

Several factors influence the spread and transmission of the infection from index patient to the household contacts. These include intensity (intimacy) and duration of contact with the index case, airflow (ventilation) in the accommodation, number of occupants, social structure, nutritional and vaccine status of contact, and genotype of the causative agent. All these parameters were studied in present study and are presented in tables (**1** **,** **3** **&** **4**) (except nutritional status). An interesting finding of the study was that very few spouses got secondary tuberculosis. This can purely be explained on the basis of social structure. In Northern India, wife-husband relationship, even though expected to be the closest relation, husbands will rarely share their illnesses with wives, rather they will prefer to share with their mothers and if mother is not alive with their friends. Also in a combined family, a bride will always keep her face covered with veil (*ghoonghat*). Moreover, in India deep kissing is not a norm for married couples in such a conservative society. Probably combination of these factors could be responsible for unexpectedly low transmission between the spouses (**Table 1** **,** **3** **&** **4**). Therefore, our findings may be used as a baseline data and will be beneficial in carrying out further contact investigations which should include some additional parameters such as nutrition especially the micronutrients, co-morbidities and other co-infections.

## Conclusion

On the basis of our study results, we can suggest that household contacts of index PTB cases carry a high risk of being diseased with TB. Through household transmission, the disease can get manifested in household contact during the active disease in index patient or even after 4–24 months of successfully treating the index patient. However, most of the secondary tuberculosis cases in a household well develop within first four months of the active disease in the index patient. Therefore, timely and pro-active screening of the household contacts could be a very effective tool to break the transmission cycle of the disease and to achieve the goal of “TB-free” India. Though females had higher risk of acquiring incident secondary tuberculosis in a household setting, fatality was significantly lower in this gender. Our results also indicate that those who were not vaccinated with BCG in childhood carried a significantly higher risk of acquiring TB.

## Materials and Methods

### Study setup

The study was initiated with the approval of the institutional ethics committee of AIIMS and written informed consent of all index and contact cases were obtained prior to enrollment. In case of children, written informed consent from their parent/guardian was taken for inclusion in the study. In this study, we have recruited open index cases with their household contacts between 2007 and 2009. All contacts were followed for 2 years. The study completed in 2011. After approval of the study by the institutional ethics committee and central TB control division of Government of India, we approached the designated microscopy centers (DMCs) of the south Delhi to identify the smear positive patients diagnosed within last two weeks. The recruitment was done using the standard inclusion criteria, as per the Centre for Disease Control (CDC), American Thoracic Society (ATS) and RNTCP guidelines in collaboration with DMCs and DOTS centers of south Delhi region (Dakshinpuri, Safdarjung hospital, Khanpur and Malaviya Nagar Colony Hospital). All the sputum smear positive patients (508) were contacted at their residence as registered at the respective DMCs. After written consent, their sputum samples collected and their household contacts (1792) were recruited. Clinical and vaccine details of both index patients and household contacts were noted. Of the 508 index patients 58 had given wrong address and 18 had no regular household contacts and thus these were excluded. Similarly out of 1792 household contacts 184 were having wrong address so they were also excluded from the study.

The study was performed at the Tuberculosis Research Laboratory, Division of Clinical Microbiology and Molecular Medicine, Department of Laboratory Medicine, All India Institute of Medical Sciences (AIIMS), New Delhi, which is an accredited laboratory. It is a tertiary care teaching hospital with 2500 bed capacity.

### Case definitions

A household was defined as a group of people living within one residence that lives and eats together and identified a head of family who makes decisions for the household. An index case was defined as the first AFB smear positive tuberculosis case identified in the household and has at least one household contact. A household contact was defined as an individual who had resided in the household for at least seven consecutive days during the three months prior to the diagnosis of tuberculosis with the index case. Household contacts included their family members, close friends, workplace contacts.

Secondary tuberculosis cases were defined as tuberculosis cases among household contacts of the index cases. These cases were classified in two types; as co-prevalent or incident TB cases. Co-prevalent tuberculosis cases were defined when the active TB was detected at the time of recruitment, while incident cases were defined when active tuberculosis was undetectable at the time of the baseline recruitment of household investigation but could be detected during the follow-up visits. Relations in the family were categorized into 3; the first degree relation included father, mother, brother and sister while in-laws and other relatives (other than spouse) were considered as second degree relatives. The clinical and demographic details of all index cases and their contacts were recorded in a prescribed format, 5 TU PPD injected, as per standard guidelines and the sputum samples from them were obtained by the field assistants and transported to the laboratory, who underwent specialized training for the purpose. The blood samples were also collected and tested for anti-mycobacterial antibodies.

### Measurements of exposure to TB and other patient characteristics

All the index cases were diagnosed on the basis of suggestive signs and symptoms, confirmed with either presence of tuberculosis bacilli on Ziehl-Neelsen (ZN) staining of sputum or mycobacterial culture in liquid medium. They were thoroughly medically evaluated by chest clinic officers. After the enrollment, the index and their contacts were followed up at regular interval of 4 months upto 24 months to detect active tuberculosis. As a standard national policy all the index cases were treated with standard DOTS regimen by respective area DOTS centers. All the details were kept confidential by the investigator and preserved for future data mining.

The household environment was evaluated for residence type, number of people in the household, number of habitable rooms. It was also noted if the rooms were having proper ventilation or open area. On the basis of above points the environment was categorized into two categories. (1) Clean: Had proper ventilation, proper sanitation, and if minimum number of rooms was two. (2) Congested: Poor ventilation, poor sanitation and if the number of room was only one.

### PPD testing

PPD testing was done on recruited subjects as described elsewhere [29]. Briefly; Tuberculin skin test (TST) was carried out by intradermally injecting 5TU (Span Diagnostics Ltd, India) purified protein derivative (PPD) into the volar surface of the forearm. While injecting PPD it was ensured that level of tuberculin syringe needle was facing upward so that a pale elevation of the skin (a wheal), 6 to 10 mm in diameter, was formed. The subjects were instructed not to apply any soap/detergent or wash the area to avoid itching and scratching for the next 48 hours. The injection site was encircled by permanent marker and reaction induration (palpable, raised, hardened area or swelling) was measured in millimeter (mm) after 48–72 hours.

### Bacteriological examination

Sputum samples were processed by NALC – NaOH method (modified Petroff's method) [31] and smear was made for AFB staining by ZN method and graded as per RNTCP guidelines [32]. Processed samples were inoculated on Lowenstein Jensen (LJ) slants as well as in automated BACTEC MGIT 960™ [Becton and Dickinson, USA]. The inoculated LJ medium slants were incubated at 37°C up to 8 weeks and screened for growth every week while MGIT960™ tubes were incubated in the MGIT 960 automated system and monitored by the system upto six weeks.

### Statistical analysis

Statistical analysis was carried out using STATA 11.0 (College station, Texas, USA). Data were described as numbers (%) or mean ± SD, as appropriate. The distribution of demographic and clinical characteristics among Index cases, co-prevalent and incident cases was described using frequency and percentage (%). To find out the factors associated with co-prevalent and incident cases, univariate followed by multivariate logistic regression analysis was used. The results were reported as odds ratio and its 95% confidence interval. The Hosmer-Lemeshow test and area under the receiver operating characteristics (ROC) curve was used to find the goodness of fit of the model. The p-value <0.05 was considered statistically significant. The serological findings are already published [33] and not covered in this paper for brevity.
